# Alkaliphiles

**Published:** 2004-04-01

**Authors:** Koki Horikoshi

**Affiliations:** Deep Star Project, Japan Marine Science and Technology Center (JAMSTEC), 2-15, Natsushima, Yokosuka, Kanagawa 237-0061

**Keywords:** Alkaliphiles, alkaliphily, alkaline enzymes, alkaline proteases, alkaline amylases, alkaline cellulases

## Abstract

The term alkaliphile is used for microorganisms that grow optimally or very well at pH values above 9, but cannot grow or grow only slowly at the near neutral pH value of 6.5. Alkaliphiles include prokaryotes, eukaryotes, and archaea. Alkaliphiles can be isolated from normal environments such as garden soil, although viable counts of alkaliphiles are higher in samples from alkaline environments. The cell surface plays a key role in keeping the intracellular pH value in the range between 7 and 8.5, allowing alkaliphiles to thrive in alkaline environments. Alkaliphiles have made a great impact in industrial applications. Biological detergents contain alkaline enzymes, such as alkaline cellulases and/or alkaline proteases that have been produced from alkaliphiles. Another important application is the industrial production of cyclodextrin with alkaline cyclomaltodextrin glucanotransferase. This enzyme reduced the production cost and paved the way for cyclodextrin use in large quantities in foodstuffs, chemicals and pharmaceuticals. It has also been reported that alkali-treated wood pulp could be biologically bleached by xylanases produced by alkaliphiles.

## Introduction

**Fig. 1:**
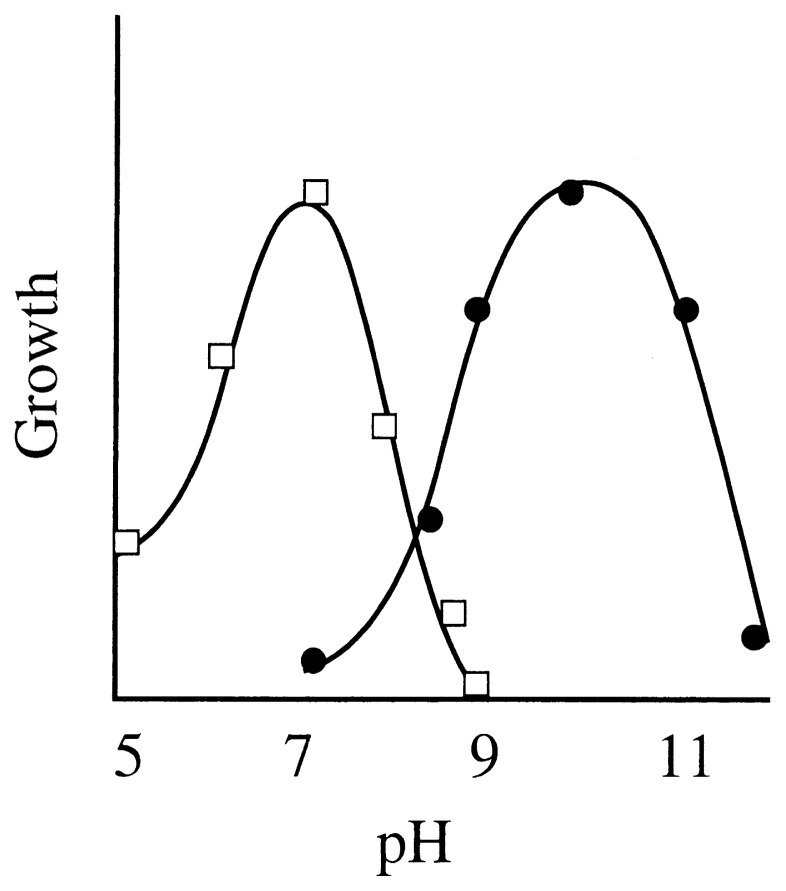
The pH dependency of alkaliphilic microorganisms. Typical growth-pH dependency of neutrophilic and alkaliphilic bacteria are shown with squares and closed circles, respectively.

The term alkaliphile is used for microorganisms that grow optimally or very well at pH values above 9, often between 10 and 12, but cannot grow or grow only slowly at the near neutral pH value of 6.5 (Fig. 1).

The discovery of alkaliphiles was fairly recent. Few scientific papers on the topic could be found, when the author started experiments on alkaliphilic bacteria in 1968. The use of alkaliphilic microorganisms has a long history in Japan, since from ancient times, indigo has been naturally reduced under alkaline conditions in the presence of sodium carbonate. Indigo from indigo leaves is reduced by particular bacteria that grow under these highly alkaline conditions in a traditional process called “indigo fermentation.” The most important factor in this process is the control of the pH value. Formerly indigo reduction was controlled only by the skill of the craftsman. Microbiological studies, however, were not conducted until the rediscovery of these alkaliphiles by the author.1) Alkaliphiles remained little more than interesting biological curiosities and at that time no further industrial application was attempted, or contemplated.

Since then, a great number of alkaliphilic microorganisms have been isolated and purified many alkaline enzymes from them. The first paper concerning an alkaline protease was published in 1971.1) Over the past three decades, our studies have focused on the enzymology, physiology, ecology, taxonomy, molecular biology, and genetics of these isolates to establish a new microbiology of alkaliphilic microorganisms. Industrial applications of these microorganisms have also been investigated extensively, and some enzymes, such as alkaline protease, alkaline amylases, and alkaline cellulases, have been put to use on an industrial scale. Alkaliphiles have clearly evolved large amounts of genetic information and exhibit an ability in their genes to cope with particular environments, so their genes are a potentially valuable source of information. Recently, the complete genomes of *Bacillus halodurans* C-125 and *Oceanobacillus iheyensis* were sequenced to obtain more information on the molecular basis of alkaliphily and on industrial applications. In this review, cell structures, genetical aspects of alkaliphily and novel alkaline enzymes will be discussed.

## Distribution and isolation of alkaliphiles

Alkaliphiles consist of two main physiological groups of microorganisms; alkaliphiles and haloalkaliphiles. Alkaliphiles require an alkaline pH of 9 or more for their growth and have an optimal growth pH of around 10, whereas, haloalkaliphiles require both an alkaline pH (>pH 9) and high salinity (up to 33% NaCl w/v). Alkaliphiles have been isolated mainly from neutral environments, sometimes even from acidic soil samples and feces. Haloalkaliphiles have been mainly found in extremely alkaline saline environments, such as the Rift Valley lakes of East Africa and the western soda lakes of the U.S.A.

## Alkaliphiles

### Aerobic alkaliphiles

**Fig. 2:**
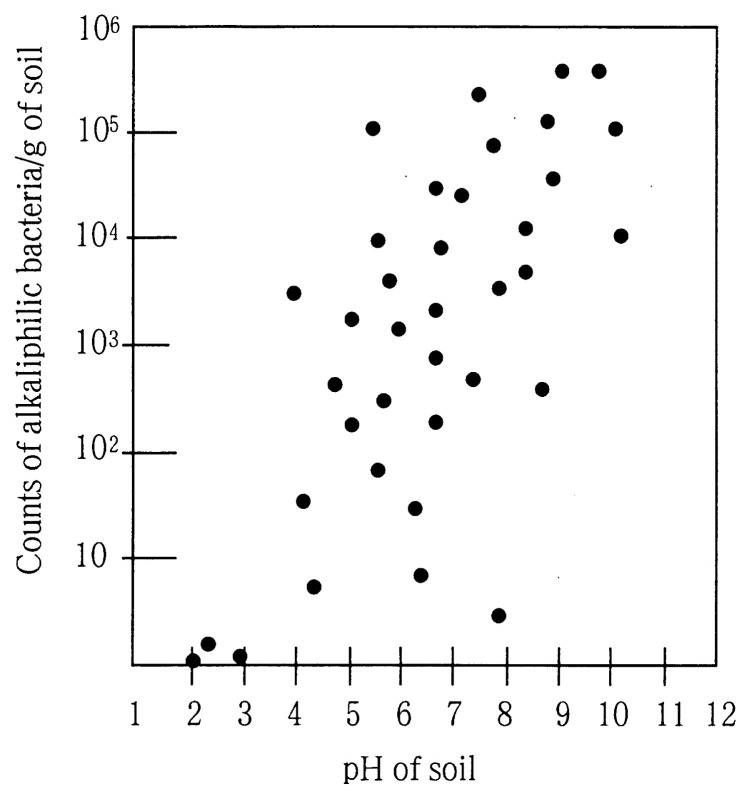
Distribution of alkaliphilic microorganisms in various pH environments.

Alkaliphilic microorganisms co-exist with neutrophilic microorganisms, as well as occupying specific extreme environments in nature. Figure 2 illustrates the relationship between the numbers of isolated alkaliphilic microorganisms and the pH of the sample origin. In order to isolate alkaliphiles, alkaline media have to be used. Sodium carbonate is generally used to adjust the pH to around 10, because almost all alkaliphiles usually require at least some sodium ions. Table I shows an alkaline medium suitable for their isolation. The frequency of alkaliphilic microorganisms in neutral “ordinary” soil samples such as garden soil has been shown to be 10^2^–10^5^/g of soil, which corresponds to 1/10 – 1/100 of the population of the neutrophilic microorganisms.2) Recent author’s studies3) show that alkaliphilic bacteria have also been found in deep-sea sediments collected from the Mariana Trench (depths as deep as the 10,898 m). Many different kinds of alkaliphilic microorganisms have been isolated from a variety of environments including bacteria belonging to the genera *Bacillus, Micrococcus, Pseudomonas* and *Streptomyces,* and eukaryotes, such as yeasts and filamentous fungi.2)–5)

### Anaerobic alkaliphiles

The first brief report on anaerobic alkaliphiles was communicated by Niimura *et al*.6) Subsequently, many anaerobic sporeforming alkaliphiles have been isolated by conventional procedures, and a few applications have been studied: Podkovyrov and Zeikus recorded the isolation and purification of a cyclomatodextrin glucanotransferase from *Clostridium thermohydrosulfuricum*-39E.7) Wiegel and colleagues 8),9) have isolated and characterized a range of thermophilic anaerobic alkaliphiles. Nine moderately alkalitolerant thermophilic bacteria with similar properties were isolated from water and soil samples obtained from Yellowstone National Park. One of eight strains represent a new genus and species, *Anaerobranca horikoshii*. An outstanding review on anaerobic alkalithermophiles has recently been reported by Wiegel.10)

### Haloalkaliphiles

The most remarkable examples of naturally occurring alkaline environments are soda deserts and soda lakes. Extremely alkaline lakes, for example, Lake Magadi in Kenya and the Wadi Natrun in Egypt, are probably the most stable highly alkaline environments on Earth, at a consistent pH of 10.5 to 12.0 depending on the site. Many organisms isolated from alkaline and highly saline environments such as soda lakes also require high salinity, which is achieved by adding NaCl to the isolation medium. In particular, hypersaline soda lakes are populated by alkaliphilic representatives of halophilic archaea.

## Specific physiological features of alkaliphiles

### Internal pH

Most alkaliphiles have an optimal growth pH at around 10, which is the most significant difference from well-investigated neutrophilic microorganisms. Therefore, the question arises as to how these alkaliphilic microorganisms can grow in such an extreme environment. Internal cytoplasmic pH of alkaliphiles can be estimated from the optimal pH of intracellular enzymes. For example, ***α***-galactosidase from an alkaliphile, *Micrococcus* sp. strain 31–2 had its optimal catalytic pH at 7.5, suggesting that the internal pH is around neutral. Furthermore, the cell-free protein synthesis systems from alkaliphiles optimally incorporate amino acids into protein at pH 8.2–8.5, only 0.5 pH units higher than that of neutrophilic *B. subtilis*.11)

Another method to estimate internal pH is to measure the inside and outside distribution of weak bases in cells, which are not actively transported by cells. The internal pH was maintained at around 8, despite a high external pH (8–11) as shown in Table II.2),12)–14) Therefore, one of the key features in alkaliphily is associated with the cell surface, which discriminates and maintains the intracellular neutral environment separate from the extracellular alkaline environment.

### Cell walls. Acidic polymers in the cell walls

Since the protoplasts of alkaliphilic *Bacillus* strains lose their stability in alkaline environments, it has been suggested that the cell wall may play a key role in protecting the cell from alkaline environments. Components of the cell walls of several alkaliphilic *Bacillus* strains have been investigated in comparison with those of the neutrophilic *B. subtilis*. In addition to peptidoglycan, alkaliphilic *Bacillus* strains contain certain acidic polymers, such as galacturonic acid, gluconic acid, glutamic acid, aspartic acid and phosphoric acid.15) The negative charges on the acidic non-peptidoglycan components keep intracellular pH value around 9, and as a consequence, assist cells to grow in alkaline environments as described below.

### Mechanisms of cytoplasmic pH regulation by cell walls

**Fig. 3:**
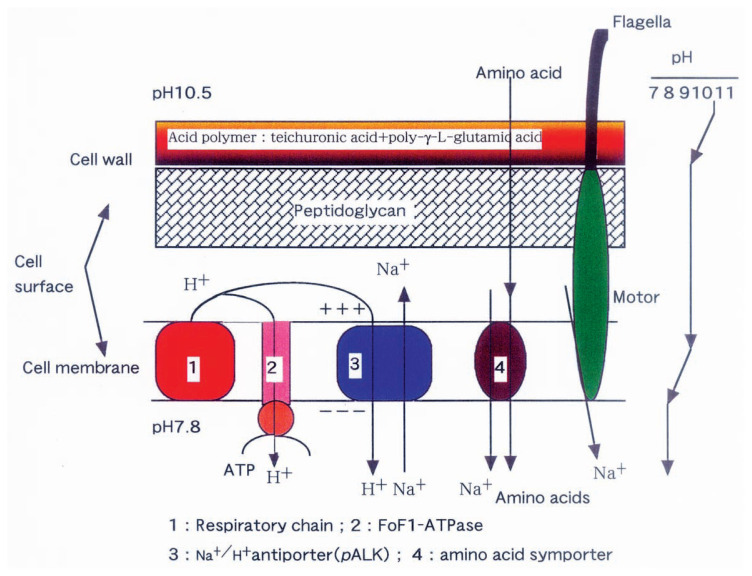
A schematic representation of cytoplasmic pH regulation.

The cells have two barriers to reduce pH values from 10.5 to 8 (Fig. 3).

Cell walls of alkaliphilic *Bacillus* species containing acidic polymers as described above function as a negatively charged matrix and reduce the pH value at the cell surface. The surface of the plasma membrane must presumably be kept below pH 9, because the plasma membrane is very unstable at alkaline pH values much below the pH optimum for growth.

It is reported that the amount of acidic polymers increases during growth under alkaline pH conditions. 16) A pH-homeostatic mechanism for alkaliphilic bacteria has been proposed taking the important role of anionic polymer layer in their cell walls into account. Donnan equilibria in the bacterial cell walls were calculated to estimate the pH values inside the polymer layer of the cell walls when the outer aqueous solution is in alkaline nature. The fixed charge concentration in the polymer layer was estimated to be 2–5 mol/l from the data reported for Gram-positive bacteria, particularly for an alkaliphilic bacterium *Bacillus halodurans* C-125. According to Tsujii’s calculation,17) the pH values estimated to exist inside a polymer layer (cell wall) are more acidic than those of the surrounding environment by 1–1.5 U. Therefore, teichuronopeptide (TupA) which is an acidic polymer of poly-*γ*-l-glutamate and polyglucuronic acid, is one of the important components in the cell wall of the alkaliphilic *B. halodurans*18) and actually contributes to the regulation of pH homeostasis in the cytoplasm.

Recently, whole genome of another alkaliphilic *Bacillus* strain, *Oceanobacillus iheyensis* was analyzed. 19) The putative protein (OB2920) showing significant similarity to the *tupA* gene product involved in TUP biosynthesis in *B. halodurans* is only shared between the two alkaliphiles. No homolog of *tupA* has been identified in any other organism, except the two alkaliphiles, thus far.

### Plasma membrane

Plasma membranes also maintain pH homeostasis by using Na^+^/H^+^ antiporter system (**ΔΨ**-dependent and **Δ**pH-dependent), K^+^/H^+^ antiporter and ATPase-driven H^+^ expulsion. Alkaliphilic microorganisms grow vigorously at pH 9–11 and require Na^+^ for growth as shown in above. According to the chemiosmotic theory, the proton-motive force in the cells is generated by the electron transport chain or by excreted H^+^ derived from adenosine triphosphate (ATP) metabolism by ATPase. H^+^ is then re-incorporated into the cells with co-transport of various substrates. In the case of Na^+^-dependent transport systems, the H^+^ is exchanged with Na^+^ by Na^+^/H^+^ antiporter systems, thus generating a sodium-motive force, which drives substrates accompanied by Na^+^ into the cells. The incorporation of a test substrate, ***α***-aminoisobutyrate (AIB) increased as the external pH shifted from 7 to 9, and the presence of sodium ion significantly enhanced the incorporation; 0.2 N NaCl produced an optimum that was 20 times the rate observed in the absence of NaCl. Other cations, including K^+^, Li^+^, NH_4_^+^, Cs^+^, and Rb^+^ showed no effect, nor did their counter-anions.20)

Recent works in several laboratories on the critical antiporters have begun to clarify the number and characteristics of the porters that support active mechanisms of pH homeostasis.21)–24)

### Genetic works on transporters

**Fig. 4:**
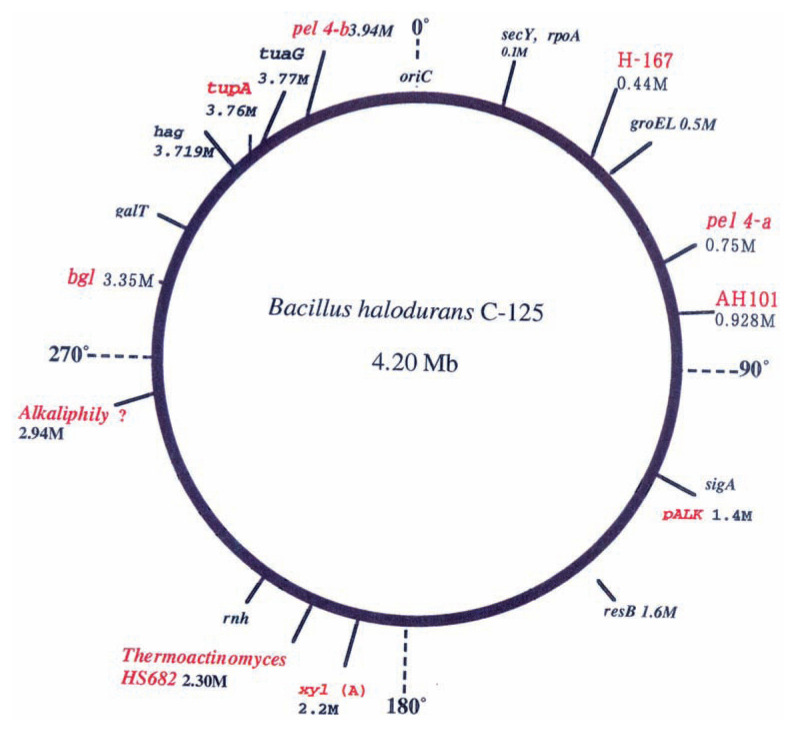
Genetic map of the chromosome of *Bacillus halodurans* C-125. The locations of several genes are indicated on the map. A dashed line indicates the approximate position of the gene.

The author’s group isolated a non-alkaliphilic mutant strain from *B. halodurans* C-125 as the host for cloning genes related to alkaliphily. A 3.7-kb parental DNA fragment (*pALK* fragment) from the parental strain restored the growth of the mutant 38154 at alkaline pH (see Fig. 4). The transformant was able to maintain an intracellular pH that was lower than the external pH and contained an electrogenic Na^+^/H^+^ antiporter driven only by **ΔΨ** (membrane potential, interior negative). These results indicate that the mutant 38154 affects, either directly or indirectly, electrogenic Na^+^/H^+^ antiporter activity. This was the first report of a DNA fragment responsible for a Na^+^/H^+^ antiporter system in the mechanism of alkaliphily. 25),26)

Krulwich and her co-workers have focused their studies on the facultative alkaliphile, *Bacillus pseudofirmus* OF4. Her work is directed toward clarification of the characteristics and energetics of membrane-associated proteins that must catalyze inward proton movements. One such protein is the Na^+^/H^+^ antiporter that enable cells to adapt to a sudden upward shift in pH and to maintain a cytoplasmic pH that is 2–2.3 units below the external pH in the most alkaline range of pH for growth. Another is the proton-translocating ATP synthase that catalyzes production of ATP under conditions in which the external proton concentration and the bulk chemiosmotic driving force are low. Three gene loci that are candidates for Na^+^/H^+^ antiporter encoding genes with roles in Na^+^-dependent pH homeostasis have been identified. All of them have homologs in *B. subtilis*, in which pH homeostasis can be carried out with either K^+^ or Na^+^. The physiological importance of one of the *B. pseudofirmus* OF4 loci, *nha*C, has been studied by targeted gene disruption, and the same approach is being extended to the others. The *atp* genes that encoded the alkaliphile’s F_1_F_0_-ATP synthase are found to have interesting motifs in areas of putative importance for proton translocation. The transformant does not exhibit growth on succinate, but shows reproducible, modest increases in the aerobic growth yields on glucose as well as membrane ATPase activity that exhibits characteristics of the alkaliphile enzyme.27) As the result of these experiments, the membrane proteins play a role in keeping the intracellular pH values in the range between 7.0–8.5.

### Whole genome sequences of alkaliphilic Bacillus strains

As shown in Fig. 4, the 4202353bp genome of *Bacillus halodurans* C-12518),19),28) contains 4066 predicted protein coding sequences (CDSs), 2141 (52.7%) of which have functional assignments, 1182 (29%) of which are conserved CDSs with unknown function and 743 (18. 3%) of which have no match to any protein database. The *B. halodurans* genome contains 112 transposase genes, indicating that transposases have played an important evolutionary role in horizontal gene transfer and also in internal genetic rearrangement in the genome. Strain C-125 lacks some of the necessary genes for competence, such as *comS*, *srfA* and *rapC*, supporting the fact that competence has not been demonstrated experimentally in C-125. There is no paralog of *tupA*, and a cluster of poly-*γ* -l-glutamate synthesizing enzyme gene encoding teichuronopeptide synthesizing enzyme, which contributes to alkaliphily, in the C-125 genome. And an ortholog of *tupA* cannot be found in the *B. subtilis* genome. Out of 11 sigma factors which belong to the extracytoplasmic function family, 10 are unique to *B. halodurans*, suggesting that they may have a role in the special mechanism of adaptation to an alkaline environment. Author’s colleague, Takami *et al*. determined another whole genome sequence of *Oceanobacillus iheyensis* HTE83119) which is an alkaliphilic and extremely halotolerant *Bacillus*-related species isolated from deep-sea sediment. They reported the complete genome sequence of HTE831 along with analyses of genes required for adaptation to highly alkaline and saline environments. The genome consists of 3.6 Mb, encoding proteins such as potentially associated with roles in regulation of intracellular osmotic pressure and pH homeostasis. The genes encoding the teichuronopeptide synthesizing enzymes that are responsible to alkaliphily were also discovered based on comparative analysis with *B. halodurans*.

Although analysis is still in progress, several open reading frames for Na^+^/H^+^ antiporters that may have roles in pH homeostasis have been detected.

## Alkaline enzymes

Studies of alkaliphiles have led to the discovery of many types of enzymes that exhibit interesting properties. The first report concerning an alkaline enzyme published in 1971 described an alkaline protease produced by *Bacillus clausii* No. 221.1) More than 35 new enzymes have been isolated and purified in the author’s laboratory. Some of these have been produced on an industrial scale.

### 1) Alkaline protease

**Fig. 5:**
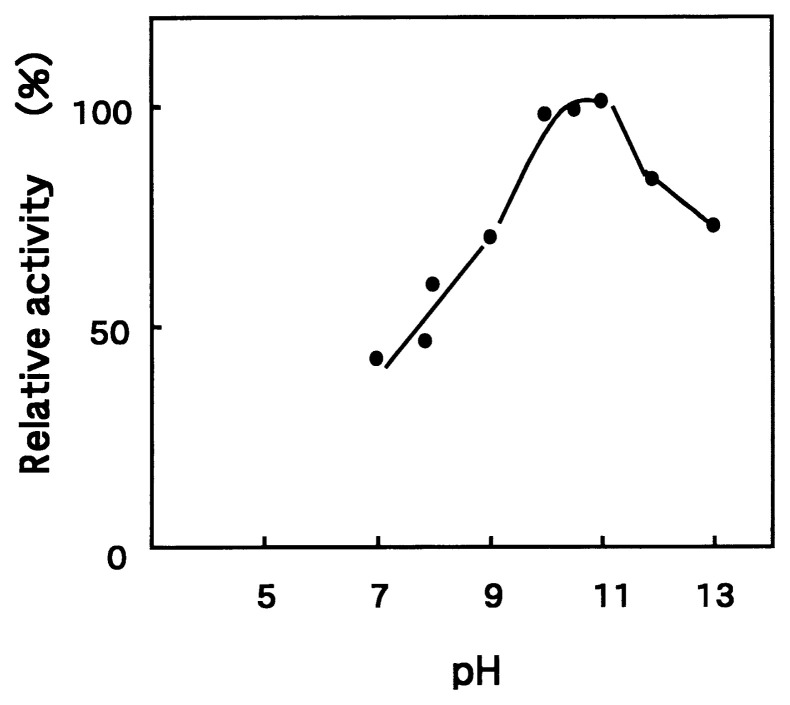
Effect of pH on protease activity.

In 1971, Horikoshi1) reported the production of an extracellular alkaline serine protease from alkaliphilic *Bacillus clausii* No. 221. This strain, isolated from soil, produced large amounts of alkaline protease that differed from the subtilisin group. The optimum pH of the purified enzyme was 11.5 with 75% of the activity maintained at pH 13.0 (Fig. 5). The addition of a 5 mM solution of calcium ions was reflected in a 70% increase in activity at the optimum temperature (60 °C). Subsequently many alkaliphilic *Bacillus* strains were reported which also produced an alkaline protease.29)

Takami *et al*.30) isolated a new alkaline protease from alkaliphilic *Bacillus halodurans* No. AH-101. The enzyme was most active toward casein at pH 12–13 and stable under 10 min incubation at 60 °C and pH 5–13. The optimum temperature was about 80 °C in the presence of 5 mM calcium ion. The alkaline protease showed a higher hydrolyzing activity against insoluble fibrous natural proteins such as elastin and keratin in comparison with subtilisins and proteinase K.

Ito and his colleagues isolated and purified an alkaline protease from alkaliphilic *Bacillus* strains suitable for use in laundry detergents.29),31) Shirai *et al.* studied the crystal structure of the M-protease of alkaliphilic *Bacillus* sp. KSM-K16 by X-ray analysis to understand the alkaline adaptation mechanism of the enzyme.32) This analysis revealed a decrease in the number of negatively charged amino acids (aspartic acid and glutamic acid) and lysine residues, and an increase in arginine and neutral hydrophilic amino acids (histidine, asparagine and glutamine) residues during the course of adaptation.

#### Other proteases

Several alkaline proteases produced by alkaliphilic actinomycetes have been reported. Tsuchiya *et al*.33) isolated thermostable alkaline protease from alkaliphilic *Thermoactinomyces* sp. HS682. The protease had the maximum proteolytic activity around pH 11.0 and at 70 °C. In the presence of Ca^+2^ ions, maximum activity was observed at 80 °C.

Extracellular proteolytic activity was also detected in the haloalkaliphilic archaeon *Natronococcus occultus* as the culture reached the stationary growth phase.34) Optimal conditions for activity were attained at 60 °C and 1–2 M NaCl or KCl. Gelatin zymography in the presence of 4 M betaine revealed a complex pattern of active species with apparent molecular masses ranging from 50 to 120 kDa.

#### Industrial applications of alkaline proteases. Detergent additives

The main industrial application of alkaliphilic enzymes is in the detergent industry, and detergent enzymes account for approximately 30% of total worldwide enzyme production. All most of these are produced by alkaliphilic bacteria. However, many alkaline proteases have been produced by alkaliphilic *Bacillus* strains and are commercially available from companies.

#### Dehairing

Alkaline enzymes have been used in the hide-dehairing process, where dehairing is carried out at pH values between 8 and 10. These enzymes are commercially available from several companies.

### 2) Starch-degrading enzymes

The first alkaline amylase was produced in Horikoshi-II medium (Table I) by cultivating alkaliphilic *Bacillus* sp. No. A-40-2.2) Several types of alkaline starch degrading enzymes were observed. No alkaline amylases produced by neutrophilic microorganisms have so far been reported. Recent studies revealed that the starch-degrading enzymes ***α***-amylase and cyclodextrin glycosyltransferase (CGTase) are functionally and structurally closely related.

#### *α*-Amylases of alkaliphilic Bacillus strains

The production of the alkaline amylase was first achieved in an alkaliphilic *Bacillus* species strain No. A-40-2 (ATCC21592) that was selected from about 300 colonies of bacteria grown in Horikoshi-II medium.2) The enzyme is most active at pH 10.0–10.5 and retains 50% of its activity between pH 9.0 and 11.5. The enzyme is not inhibited by 10 mM EDTA at 30 °C, and is completely inactivated by 8 M urea. The enzyme can hydrolyze 70% of starch to yield glucose, maltose, and maltotriose and the enzyme is a type of saccharifying ***α***- amylase. Subsequently, considerable diversity of ***α***- amylases has been reported: Boyer *et al*.35) reported an alkaline amylase in the strain NRRL B-3881, which was the second report of an alkaline amylase. The B-3881 amylase had its optimum pH for enzyme action at 9.2. The enzyme yields maltose, maltotriose, and a small amount of glucose and maltotetraose, all of which have a ***β***-configuration.

Igarashi *et al*. isolated a novel liquefying ***α***-amylase (LAMY) from cultures of an alkaliphilic *Bacillus* isolate, KSM-1378.36) The enzyme had a pH optimum of 8.0 to 8.5 and displayed maximum activity at 55 °C. The structural gene for the amylase contained a single open reading frame 1,548 bp in length, corresponding to 516 amino acids that included a signal peptide of 31 amino acids. The four conserved regions were found in the deduced amino acid sequence. Essentially, the sequence of LAMY was consistent with the tertiary structures of reported amylolytic enzymes, which are composed of domains A, B, and C. Furthermore, they37) improved the thermostability of the amylase by deleting an arginineglycine residue in that molecule.

#### Other *α*-amylases

Kimura and Horikoshi 38) isolated a number of starch-degrading psychrotrophic microorganisms from the environments. A haloalkaliphilic Natronococcus sp. strain Ah-36, produced an extracellular maltotriose-forming amylase.39) The amylase exhibited maximal activity at pH 8.7 and 55 °C in the presence of 2.5 M NaCl, then they have cloned this ***α***- amylase and expressed it in Haloferax volcanii.

#### Cyclomaltodextrin glucanotransferases (CGTase)

Nakamura and Horikoshi discovered many alkaliphilic *Bacillus* strains producing cyclomaltodextrin glucanotransferases. The crude enzyme of *Bacillus* sp. No. 38-2 was a mixture of three enzymes: acid CGTase having optimum pH for enzyme action at 4.6, neutral CGTase at 7.0, and alkaline CGTase at 8.5.40) In 1975, Matsuzawa *et al*. established the industrial production of cyclodextrin by using crude CGTase of *Bacillus* sp. 38-2.41) Since then, many alkaliphilic microorganisms producing CGTases have been reported. The CGTase produced predominantly ***β***-cyclodextrin (***β***-CD), with minor amounts of ***α***- and ***γ***-CDs. Recently, Parsiegla *et al*. reported that mutation of *Bacillus circulans* strain no. 8 was effective in increasing the ***γ***-cyclodextrin production.

Yamane’s group has extensively investigated molecular structure of the CGTase of alkaliphilic *Bacillus* sp. 1011. Kimura *et al*. isolated the enzyme, purified and cloned its gene.43) The enzyme, consisting of 686 amino acid residues, was crystallized and subjected to X-ray analysis. The molecule consists of five domains, designated A-E, and its backbone structure is similar to the structure of other bacterial CGTases. The molecule has two calcium binding sites where calcium ions are coordinated by seven ligands, forming a distorted pentagonal bipyramid. Three histidine residues in the active center of CGTase participate in the stabilization of the transition state. His-327 is especially important for catalysis over the alkaline pH range.44) Three-dimensional structures of cyclodextrin glucanotransferases (CGTases) have revealed that four aromatic residues, which are highly conserved among CGTases but not in ***α***-amylases, are located in the active center.

#### Industrial production of cyclodextrins

In 1969, Corn Products International Co., U.S.A., began producing ***β***-CD using *B. macerans* CGTase. Teijin Ltd. in Japan also produced ***β***-CD using the *B. macerans* enzyme in a pilot plant. But there were several serious problems in both production processes. (1) The yield of CD from starch was not high. (2) Toxic organic solvents such as trichloroethylene, bromobenzene, and toluene were used to precipitate CD owing to the low conversion rate. The use of CGTase of alkaliphilic *Bacillus* sp. No. 38-2 overcame all these weak points and led to the mass-production of crystalline ***α***-, ***β***-, ***γ***-CD at low cost without using any organic solvents. The yield of CD was 85–90% from amylose and 70–80% from potato starch on a laboratory scale. Owing to the high conversion rate, CDs could be directly crystallized from the hydrolyzate of starch without the addition of organic solvents.41) These simple methods developed reduced the cost of ***β***- CD from 199,000 yen to 1,000 yen/kg, and that of ***α***-CD to within 3,000 yen/kg. This has paved the way for its use in large quantities in foodstuffs, chemicals, and pharmaceuticals. Several other processes to produce CDs have been reported after Matsuzawa’s original paper. CDs production plants in the world are essentially operated by almost same as those of author’s process.

#### Pullulanases

In 1975, Nakamura *et al*. discovered an alkaline pullulanase of *B. halodurans* No. 202-1.45) The enzyme has an optimum pH for enzyme action at 8.5–9.0 and is stable for 24 h at pH 6.5–11.0 at 4 °C. The enzyme is most active at 55 °C, and is stable up to 50 °C for 15 min in the absence of substrate. Kelly *et al*.46) reported that alkaliphilic *Bacillus halodurans* No. A-59 produced three enzymes, ***α***-amylase, pullulanase and ***α***- glucosidase in culture broth. These three enzymes were separately produced and the levels of ***α***-glucosidase and pullulanase reached maxima after 24-h cultivation at the initial pH 9.7. Although this pullulanase was not purified, the indicated pH optimum was at 7.0.

Two highly alkaliphilic pullulanase-producing bacteria were isolated from Korean soils.47) The two isolates were extremely alkaliphilic since bacterial growth and enzyme production occurred at pH values ranging from pH 6.0 to 12.0 for *Micrococcus* sp. Y-1 and pH 6.0 to 10.0 for *Bacillus* sp. S-1. The enzyme displayed a temperature optimum of around 60 °C and a pH optimum of around pH 9.0.

In screening alkaline cellulases for detergent additives, Ito’s group isolated a novel alkaline pullulanase from alkaliphilic *Bacillus* sp. KSM-1876, which was identified as a relative of *Bacillus circulans*.48),49) The enzyme had an optimum pH for enzyme action of around 10.0–10.5. This enzyme is a good candidate for use as an additive to dishwashing detergents. They then found another alkaline amylopullulanase from alkaliphilic *Bacillus* sp. KSM-1378. This enzyme efficiently hydrolyzed ***α***-1,4 and the ***α***-1,6 linkages at alkaline pH values. The kinetic studies revealed two independent active sites for the ***α***-1,4 and ***α***-1,6 hydrolytic reactions. Incubation of the enzyme at 40 °C and pH 9.0 caused complete inactivation of the amylase activity within 4 days, but the pullulanase activity remained at the original level under the same conditions. This alkaline amylopullulanase can, therefore, be considered to be a “two-headed” enzyme molecule. Limited proteolysis with papain also revealed that the ***α***-1,6 and ***α***-1,4 hydrolytic activities were associated with two different active sites. Furthermore, amino acid sequence and molecular structure analysis of the enzyme showed two different active sites. The enzyme was observed by transmission electron microscopy; it appeared to be a bent dumbbell-like molecule with a diameter of approximately 25 nm.

In some food industries, enzymes showing activity at lower temperatures have been requested for food processing. Psychrotrophic bacteria are though to be potential producers of these enzymes. Kimura and Horikoshi50) reported that an alkalipsychrotrophic strain, *Micrococcus* sp. 207, extracellularly produced amylase and pullulanase. The pullulanase of *Micrococcus* sp. 207 was purified to an electrophoretically homogeneous state by conventional ways. The purified enzyme was free of ***α***-amylase activity. The enzyme had a pH optimum at 7.5–8.0 and was relatively thermostable (stable up to 45 °C). The enzyme could hydrolyze the ***α***-1,6-linkages of amylopectins, glycogens and pullulan. Although many alkaline pullulanases have been reported as described above, no industrial application has been developed yet.

### 3) Cellulases. Alkaline cellulases of alkaliphilic Bacillus strains

Commercially available cellulases display optimum activity over a pH range from 4 to 6. No enzyme with an alkaline optimum pH for activity (pH 10 or higher) had been reported before the rediscovery of alkaliphiles. Horikoshi and his colleagues found bacterial isolates (*Bacillus* sp. No. N-4 and No.1139) producing extracellular alkaline carboxymethylcellulases (CMCases).51) One of these, alkaliphilic *Bacillus* sp. No. N-4 (ATCC21833) produced multiple CMCases that were active over a broad pH range (pH 5–10). Sashihara *et al*. cloned the cellulase genes of *Bacillus* sp. No. N-4 in *Escherichia coli* HB101. Several cellulase-producing clones that have different DNA sequences were obtained. Another bacterium, *Bacillus* sp. No. 1139, produced one CMCase, which was purified and shown to have optimum pH for activity at pH 9.0. The enzyme was stable over the range of pH 6–11 (24 h at 4 °C and up to 40 °C for 10 min).

#### Cellulases as laundry detergent additives

The discovery of alkaline cellulases created a new industrial application of cellulase as a laundry detergent additive. Ito (personal communication) mixed alkaline cellulases with laundry detergents and studied the washing effect by washing cotton underwear. The best results were obtained by one of the alkaline cellulases produced by an alkaliphilic *Bacillus* strain. However, the yield of enzyme was not sufficient for industrial purposes. Consequently, Ito *et al*.52) isolated an alkaliphilic *Bacillus* sp. No. KSM-635 from the soil and succeeded in producing an alkaline cellulase as a laundry detergent additive on an industrial scale.

Besides the KSM-635 enzyme, Shikata *et al*. isolated three strains, alkaliphilic *Bacillus* KSM-19, KSM-64, and KSM-520, producing alkaline cellulases for laundry detergents.53) Their activities (pH optima: 8.5–9.5) were not inhibited at all by metal ions or various components of laundry products, such as surfactants, chelating agents and proteinases. With a view to increasing industrial production, they over expressed alkaline cellulase of alkaliphilic *Bacillus* sp. KSM-64 by using *Bacillus subtilis* harboring their vector pHSP64. By this process they produced 30 g of alkaline cellulase in one liter. After the discovery of the industrial application of alkaline cellulase as a detergent additive, many microbiologists have extensively studied alkaline cellulases.

#### Alkaline cellulases from other alkaliphiles

Damude *et al*.54) studied a semi-alkaline cellulase produced by alkaliphilic *Streptomyces* strain KSM-9. Dasilva *et al*.55) reported two alkaliphilic microorganisms, *Bacillus* sp. B38-2 and *Streptomyces* sp. S36-2. The optimum pH and temperature of the crude enzyme activities ranged from 6.0 to 7.0 at 55 °C for the *Streptomyces* and 7.0 to 8.0 at 60 °C for the *Bacillus* sp. B38-2. However, their results indicated that the properties of these enzymes were not sufficient for industrial purposes.

### 4) Alkaline lipases

Although the initial motivation for studying alkaline lipase was its application to detergents, many alkaline lipases were significantly inhibited in the presence of either alkylbenzene sulfate or dodecyl benzene sulfonate.

Watanabe *et al*.56) conducted an extensive screening for alkaline lipase producing microorganisms from soil and water samples. Two bacterial strains were selected as potent producers of alkaline lipase. These were identified as *Pseudomonas nitroreducens* nov. var. *thermotolerans* and *Ps. fragi*, respectively. The optimum pH of the two lipases was 9.5. Both enzymes were inhibited by bile salts such as sodium cholate, sodium deoxycholate, and sodium taurocholate at a concentration of 0.25%.

In 1995, a thermophilic lipase producing bacterium was isolated from a hot spring area of Yellowstone National Park.57) The organism characterized as *Bacillus* sp. grew optimally at 60–65 °C and in the pH range of 6–9. The partially purified lipase preparation had an optimum temperature of 60 °C, at an optimum pH of 9.5. It retained 100% of the original activity after being heated at 75 °C for half an hour. The enzyme was active on triglycerides containing fatty acids having a carbon chain length of C_16:0_ to C_22:0_ as well as on natural fats and oils.

### 5) Xylanases. Xylanases of alkaliphilic Bacillus strains

The first paper describing an xylanase from alkaliphilic bacteria was published in 1973 by Horikoshi and Atsukawa.58) The purified enzyme of *Bacillus* sp. No. C-59-2 exhibited a broad optimum pH ranging from 6.0 to 8.0. And, in the culture broth of *Bacillus halodurans* No. C-125, two xylanases were found.59) Xylanase A (see Fig. 4) had molecular weight of 43,000 and that of xylanase N was 16,000. Xylanase N was most active at pH 6–7 and xylanase A was most active at a pH range of 6 to 10 and had some activity at pH 12. Xylanase A gene was cloned, sequenced and expressed in *E. coli*. Four thermophilic alkaliphilic *Bacillus* stains (Wl (JCM2888), W2 (JCM2889), W3 and W4) produced xylanases.60) The pH optima for enzyme action of strains Wl and W3 was 6.0 and for strains W2 and W4 was between 6 to 7. The enzymes were stable between pH 4.5 and 10.5 at 45 °C for 1 h. The optimum temperatures of xylanases of W1 and W3 were 65 °C and those of W2 and W4 were 70 °C. The degree of hydrolysis of xylan was about 70% after 24 h incubation.

After the demonstration that alkali-treated woodpulp could be biologically bleached by xylanases instead of by the usual environmentally-damaging chemical process using chlorine, the search for thermostable alkaline xylanases has been extensive. Dey61) isolated an alkaliphilic thermophilic *Bacillus* sp. (NCIM 59) that produced two types of cellulase-free xylanase at pH 10 and 50 °C. Khasin *et al*. reported alkaliphilic *Bacillus stearothermophilus* T-6 produced an extra-cellular xylanase that was shown to optimally bleach pulp at pH 9 and 65 °C.62) Nakamura *et al*. also isolated alkaliphilic *Bacillus* sp. strains from soil produced multiple xylanases extracellularly.63),64) One of the enzymes, xylanase J, was most active at pH 9.0. The optimum temperature for the activity at pH 9.0 was around 50 °C. Then, an alkaliphilic and thermophilic *Bacillus* sp. strain TAR-1 was isolated from soil. The xylanase was most active over a pH range of 5.0 to 9.5 at 50 °C. Optimum temperatures of the crude xylanase preparation were 75 °C at pH 7.0 and 70 °C at pH 9.0. These xylanases did not act on cellulose, indicating a possible application of the enzyme in biological debleaching processes. Subsequently, many thermostable alkaline xylanases have been produced from various alkaliphiles isolated from geothermal area.

### 6) Pectinases

The first paper on alkaline endopolygalacturonase produced by alkaliphilic *Bacillus* sp. No. P-4-N was published in 1972. And Kobayashi *et al*. reported the gene for the enzyme from alkaliphilic *Bacillus* sp. strain P-4-N was cloned, sequenced, and overexpressed in *Bacillus subtilis* cells. The deduced amino acid sequence of the mature enzyme (318 amino acids, 34805 Da). The optimum pH for enzyme action was 10.0 for pectic acid.65),66) The first application of alkaline pectinase-producing bacteria in the retting of Mitsumata bast was reported by Yoshihara and Kobayashi.67) The pectic lyase (pH optimum 9.5) produced by an alkaliphilic *Bacillus* sp. No. GIR 277 has been used in improving the production of a type of Japanese paper. A new retting process produced a high-quality, nonwoody paper that was stronger than the paper produced by the conventional method. Tanabe *et al*.68) tried to develop a new waste treatment by using an alkaliphilic *Bacillus* sp. No. GIR 621-7.

### 7) Chitinase

Tsujibo *et al*.69) isolated chitinases from an alkaliphilic *Nocardiopsis albus* subsp. *prasina* OPC-131. The isolate produced two types of chitinases. The optimum pH of chi-A was pH 5.0, and that of chi-B was pH 7.0. Recently, Bhushan and Hoondl70) isolated an alkaliphilic, chitinase-producing *Bacillus* sp. BG-11. The purified chitinase exhibited a broad pH and temperature optima of 7.5–9.0 and 45 °C-55 °C, respectively. The chitinase was stable between pH 6.0–9.0 and 50 °C for more than 2 h. Ag^+^, Hg^2+^, dithiothreitol, ***β***-mercaptoethanol, glutathione, iodoacetic acid and iodoacetamide inhibited the activity up to 50%. No further works have been reported.

## Future of alkaliphiles

Since the rediscovery of alkaliphilic bacteria, more than 1,500 papers have been published on many aspects of alkaliphiles and alkaliphily. The alkaliphiles are unique microorganisms with great potential for microbiology and biotechnological exploitation. The aspects that have received the most attention in recent years include: (1) extracellular enzymes and their genetic analysis; (2) mechanisms of membrane transport and pH regulation; and (3) the taxonomy of alkaliphilic microorganisms. What will be the next line of development? It is unclear, but it may be the wider application of enzymes. Alkaline enzymes should find additional uses in various fields of industry, such as chiral synthesis, biological wood pulping, and more sophisticated enzyme detergents. Furthermore, alkaliphiles may be very good general genetic resources, for such applications as signal peptides for secretion, and promoters for hyperproduction of enzymes.

## Figures and Tables

**Table I:** Basal media for alkaliphilic microorganisms

Ingredients	Horikoshi-I (g/l)	Horikoshi-II (g/l)
Glucose	10	–
Soluble starch	–	10
Polypeptone	5	5
Yeast extract	5	5
KH_2_PO_4_	1	1
Mg_2_SO_4_ · 7H_2_O	0.2	0.2
Na_2_CO_3_	10	10
Agar	20	20

**Table II:** Intracellular pH values in alkaliphilic *Bacillus* strains at different external pH values.

Microorganisms	External pH	Internal pH
*B. alcalophilus*	8.0	8.0
	9.0	8.2
	10.0	8.6
	11.0	9.2
*B. pseudofirmus*	7.0	7.7
	9.0	8.0
	10.5	9.4
*B. halodurans*	7.0	7.3
C-125	7.5	7.4
intact cells	8.0	7.5
	8.5	7.5
	9.0	7.6
	9.5	7.8
	10.0	8.2
	10.5	8.4
	11.0	8.8
	11.5	9.7
*B. halodurans*	7.0	7.5
C-125	8.0	7.9
protoplasts	8.5	8.2
	9.0	8.4
	9.3	8.6
